# The Role of Regulated mRNA Stability in Establishing Bicoid Morphogen Gradient in *Drosophila* Embryonic Development

**DOI:** 10.1371/journal.pone.0024896

**Published:** 2011-09-16

**Authors:** Wei Liu, Mahesan Niranjan

**Affiliations:** School of Electronics and Computer Science, University of Southampton, Southampton, United Kingdom; Universidade Federal do Rio de Janeiro, Brazil

## Abstract

The Bicoid morphogen is amongst the earliest triggers of differential spatial pattern of gene expression and subsequent cell fate determination in the embryonic development of *Drosophila*. This maternally deposited morphogen is thought to diffuse in the embryo, establishing a concentration gradient which is sensed by downstream genes. In most model based analyses of this process, the translation of the *bicoid* mRNA is thought to take place at a fixed rate from the anterior pole of the embryo and a supply of the resulting protein at a constant rate is assumed. Is this process of morphogen generation a passive one as assumed in the modelling literature so far, or would available data support an alternate hypothesis that the stability of the mRNA is regulated by active processes? We introduce a model in which the stability of the maternal mRNA is regulated by being held constant for a length of time, followed by rapid degradation. With this more realistic model of the source, we have analysed three computational models of spatial morphogen propagation along the anterior-posterior axis: (a) passive diffusion modelled as a deterministic differential equation, (b) diffusion enhanced by a cytoplasmic flow term; and (c) diffusion modelled by stochastic simulation of the corresponding chemical reactions. Parameter estimation on these models by matching to publicly available data on spatio-temporal Bicoid profiles suggests strong support for regulated stability over either a constant supply rate or one where the maternal mRNA is permitted to degrade in a passive manner.

## Introduction

Differential cell fate determination in space, leading to patterning in embryonic development, is mainly thought to be caused by spatial concentration gradients of a class of molecules known as morphogens. This view, put forward by Turing [1] over 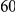 years ago, is a computational model which predicted the mechanism long before an example of it being discovered in the real world. The idea that morphogen diffuses from a localized source and provides different concentration thresholds to generate positional information was formalized by Wolpert [2] as the French flag model, which is in conjunction with an early quantitative model proposed by Crick [3].

The most definitive example, the Bicoid transcription factor, maternally deposited as mRNA molecules at the anterior pole of *Drosophila melanogaster* embryos, is translated into protein and propagates along the anterior-posterior axis, setting up a concentration gradient [4]–[6]. This, in conjunction with other similar transcription factors, regulates the establishment of the segmental structure by precise activation of downstream gap genes [7]–[9]. Many computational and experimental works have been published towards an understanding of the precision with which spatial boundaries are established and the scaling behaviour of the concentration gradients have been analysed [10]–[18]. Several computational models of Bicoid gradient formation have been published over the last three decades (reviewed by Grimm [19]). The most widely used one is the formulation of Wolpert [2] and Crick [3] published before the discovery of the role of Bicoid, in which a combination of protein synthesis, diffusion and degradation (SDD) is the underlying mechanism that derives a steady state concentration gradient. Decoding differential concentrations from such a gradient, which is spatially exponential in the steady state, and robustness properties of it are discussed in [5], [10]. Hecht *et al.* [20] propose a model, based on an additional cytoplasmic flow term, which is motivated by the argument that, with passive diffusion, the quantitative properties of the morphogen profiles establishment require higher values of diffusion constant than have been experimentally measured [12], [13].

In an alternate approach, focusing on the discrete nature of the molecular system, Wu *et al.* [21] present a probabilistic formulation, treating the embryo as a finite number of compartments, and formulating the chemical master equation for molecules making transitions between them. Because it is hard to obtain the analytical solutions of reaction diffusion master equation, numerical simulations based on Gillespie algorithm [22] are used for inference in this model. An elegant approximate inference method for such stochastic models is presented in Dewar *et al.* [23], drawing on statistical physics literature. They propose a Bayesian approach, based on formulating a Markov Jump Process, for estimating parameters from observational data, along with uncertainties in these estimates. While inference from such a system is usually achieved via stochastic simulations, the authors use approximate inference to circumvent the associated computational complexities.

Alternatives to passive deterministic diffusion from a point source at the anterior end has been considered by some recent authors. Coppey *et al.* in [24], [25] propose such a mechanism for Bicoid gradient establishment based on the idea of nuclear trapping. Their model explicitly accommodates the growth in the number of nuclei in the embryo, and the shuttling of Bicoid molecules in and out of nuclei at each cycle. This mechanism, in essence, serves as a substitute for degradation of the morphogen molecules assumed in other models. A more recent model due to Kavousanakis *et al.* [26] considers an arrangement of periodic components representing nuclei, modelling very much the same nuclear trapping aspect studied by Coppey *et al.*. Spirov *et al.* [27] propose a totally different perspective of the existence of an mRNA gradient along the A-P axis. We take some points from this particular work later in this paper. Cheung *et al.* [28] have considered Bicoid production rate as a variable, *i.e.* the quantity of maternally deposited mRNA being a function of the size of the embryo, as an explanation of morphogen gradient scaling.

While most work on the subject focuses only on the steady state properties of the exponential profile, Bergmann *et al.* [29] suggest that much of the desirable properties of this profile is also available in the pre-steady state stages of morphogen diffusion. They also provide some experimental evidence in support of this hypothesis.

Quantitative models of decoding following the establishment of a steady state profile have been considered by researchers. The complex expression of gap genes that drive segmentation along the A-P axis is studied in [30]–[34] by the construction of a gene circuit model. This body of work, closely associated with the literature on artificial neural networks, shows how dynamical properties of a nonliear network of interacting transcription factors achieves segmentation along the A-P axis by differential expressions. Remarkably, the models are able to exhibit dynamical shifts of gap gene expression peaks from posterior towards anterior. These authors mostly assume Bicoid to have a sustained exponential steady state profile throughout the analysis intervals they consider, a questionable assumption since it is precisely during this time interval that the morphogen degrades rapidly. Computational complexities of parameter estimation for such gene circuits, and the use of sophisticated evolutionary algorithms, are considered in [35].

To the best of our knowledge, all computational models mentioned above assume that the translation of maternal mRNA takes place at a constant rate at the anterior end, resulting in a constant supply of morphogen to diffuse in the system. Although mathematically convenient, in that it leads to easy closed-form solutions, this is an unrealistic assumption, for there is no need for the embryo to continue to maintain a constant supply of morphogen beyond what is needed for downstream decoding.

A particular view on this subject, supported by experimental findings, is advanced by Surdej and Jacobs-Lorena [36] who argue that the stability of the *bicoid* mRNA is regulated during development; the mRNA being held stable during the first two hours of development and rapidly killed off thereafter. Spirov *et al.* [27]'s work, proposing an mRNA spatial distribution for *bicoid* also contains further experimental evidence pointing in this direction. By fluorescence in situ hybridization (FISH) method and confocal microscopy, these authors confirm that *bicoid* mRNA disappears below detectable levels around 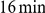 after the onset of nuclear cycle 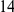 with complete mRNA degradation taking place over a time interval of 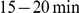.

In this paper, we pursue these observations of the regulation of stability, leading to a model of morphogen propagation in which the source supply is assumed to consist of a constant part during early development, followed by an exponential decay.

We integrate such a source model into three different models of morphogen propagation and match the resulting spatio-temporal profiles to measurements published by the FlyEx database [37], [38]. By matching the model output to FlyEx measurements, using a least squares fitting method, we infer optimal parameters of each of the models, including the time at which mRNA stability is destroyed. We also quantify the uncertainties in these estimates by constructing bootstrapped sample paths through different individual fly measurements, taken at different developmental stages. Our results show that the estimated parameters all lie in sensible ranges of values, and the decay onset time inferred from data coincides well with the experimental observations in [27], [36]. While the control of stability and translation during development have been discussed by other authors (*e.g.* see review by Cooperstock and Lipshitz [39]), these have not been included in computational models.

As such, ours is the first *in-silico* study that incorporates a novel mechanism of developmental regulation by which a morphogen gradient is established when needed, and killed off by some active processes once its task is accomplished. This is something one would naturally expect, but is ignored in three decades of modelling work on the subject.

## Results and Discussion

### *bicoid* mRNA Regulation

**Figure 1:**
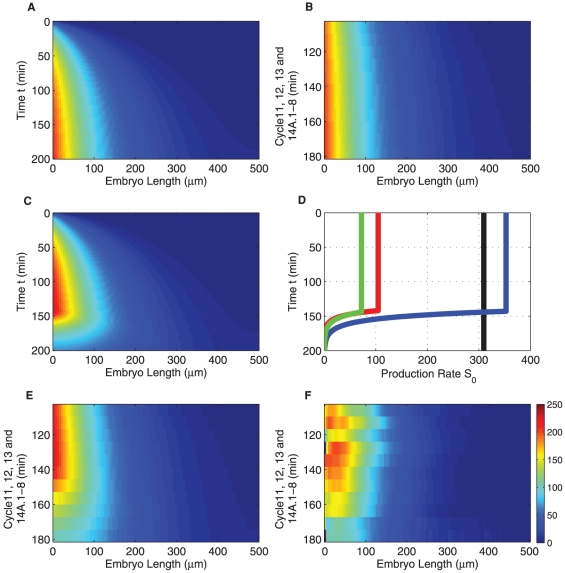
Spatio-temporal profiles of Bicoid and regulated anterior mRNA profiles inferred using three different computational models. (A) 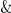 (B) spatio-temporal profiles for a conventional model that assumes a constant source (drawn over two timescales). Inferred source profiles are in shown in (D), for deterministic diffusion (blue), cytoplasmic flow (red) and the stochastic (green) models. They differ in the source amplitudes required to fit the data, but the estimated decay onset times are very close. The corresponding spatio-temporal profile is shown in (C) over the full time and space axes. (E) and (F): model output and FlyEx data in the space-time range over which optimization was carried out. Profile shown in (E) is only for the deterministic diffusion model for clarity.

We implemented *bicoid* mRNA stability regulation in Bicoid reaction diffusion systems with different computational models. Figure 1 shows various spatio-temporal profiles of Bicoid concentrations along the time and A-P axes of the embryo. Figure 1A is the profile, over the entire timescale since egg-laying in which an exponential profile is achieved and maintained as a steady state. Figure 1B is a zoomed-in version of this during nuclear cleavage cycles 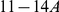, which corresponds in time to the measurements from FlyEx shown in Figure 1F. Clearly, we do not see the post-peak decay of morphogen in the embryo because it has not been modelled. Results of our novel computational diffusion model in which the source supply incorporates regulated stability are shown in Figure 1C and E, at the full and post-peak time windows respectively. We observe that the decay seen in the database is faithfully captured in Figure 1E. The corresponding source functions, inferred from data are shown in Figure 1D for all four models (including the model without mRNA regulation) considered. We see that the decay onset and the rate at which the source is rapidly decayed are in agreement for all three models considered. Equivalent results of spatio-temporal Bicoid distribution for the stochastic simulation model and the cytoplasmic flow model are given in Figures S1 and S2.

We see from Figure 1D that the modelling process correctly recovers a source function consistent with the hypothesis of regulated mRNA stability as noted from the experimental work of Surdej and Jacobs-Lorena [36]. Further support for this observation can also be found in the work of Salles *et al.* [40]. By using polymerase chain reaction (PCR) - based assay, they showed that the poly(A) tail of *bicoid* mRNA dynamically increases during the first 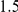 hours of development and subsequently rapidly decreases in length. As the poly(A) tail has the feature of protecting mRNA for degradation, this may be the mechanism by which stability regulation is achieved.

With mRNA regulation, the degrading Bicoid could have a contribution to the dynamic shifts in the position of gap gene expression domains which are the particular aspects of the gene regulation circuit model [20]. Bicoid as an external input in this circuit is implemented as a constant exponential function along the embryo. It is likely that the degrading Bicoid will also have a contribution towards the P-A shift of expression peaks observed by the authors.

### Parameter Estimation

For the deterministic diffusion and stochastic models, there are four parameters (diffusion constant 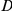, protein half life 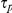, source mRNA half life 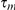 and decay onset time 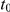). For Hecht *et al.* [20]'s flow model, there is an additional parameter, the flow velocity 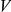. Please refer to Materials and Methods for details of model specifications and the data fitting procedure, which include exhaustive search on a grid of feasible parameter values (Table S1) for the unknowns and a closed form solution for the overall source amplitude.

Values of estimated parameters for the different models are shown in Table 1, for the regulated stability model and a model in which source mRNA is permitted to decay from time zero (unregulated). We note that parameter values estimated by the fitting procedure are in sensible ranges used by previous authors.

**Table 1:** Parameter estimation.

	Regulated Stability			Unregulated mRNA Decay		
Estimated Parameters	Diffusion	Stochastic	Flow	Diffusion	Stochastic	Flow
Diffusion constant 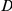 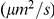	3	3	0.9	1.1	1.1	0.4
mRNA decaying onset time 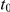 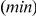	143	144	142	N/A	N/A	N/A
Bicoid proteins half-life 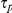 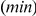	87	86	42	250	250	156
*bicoid* mRNA half-life 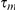 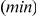	9	9	7	38	37	13
Flow velocity 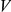 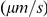	N/A	N/A	0.04	N/A	N/A	0.01
Source intensity 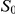	352	72	104	901	188	980

Parameter values estimated by matching model outputs to observed data from FlyEx. Least squares fitting of model outputs to FlyEx with exhaustive search for the best combination of parameters on a regular grid suggests sensible values for the mRNA decay onset time, 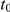, in all three models. Regulated stability corresponds to an optimized period in time during which the mRNA is held stable and translated at a constant rate, followed by rapid decay. Unregulated stability is where the mRNA is allowed to decay from the very beginning; these parameters were estimated by forcing 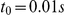 in the optimization loop.

As already seen in Figure 1, for the regulated stability estimates, there is strong agreement across the three different models with respect to the onset of source decay (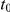), and the speed at which it is decayed (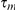), the main focus of our investigation. As noted in the Introduction, these observations confirm the experimental findings in [36] and [27]. Surdej and Jacobs-Lorena [36] argue that the mRNA is developmentally regulated, *i.e.* being held stable for up to the first two hours and then rapidly killed off in the next 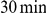. Spirov *et al.* [27] also suggest that the rapid degradation takes place over a 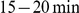 interval. The rapid decay of mRNA suggested in both these papers is consistent with half-lives of 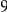, 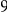 and 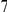 minutes inferred from our models.

We note that the diffusion constant estimated for Hecht *et al.* [20]'s cytoplasmic flow model is smaller than the other two. This is to be expected since the motivation of this model is to use cytoplasmic flow as an additional trafficking mechanism that offsets a low diffusion constant. The value we estimated for flow velocity (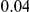 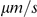) is close to what was used in [20] (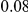 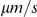), who take this estimate from observed nuclear motions. They note a 20-fold large range of possible values for this parameter, and use an average value. It is encouraging that the parameter obtained by fitting to FlyEx happens to be quite close.

The rightmost three columns of Table 1, show the parameter estimation for an unregulated source which allows for mRNA decay from time zero. This possibility is a natural expectation we need to explore, since mRNA molecules are inherently unstable. In order to match the measurements in the post-peak region, it turns out that this model not only has to amplify the source (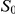) to almost ten times of the other models but also has to retain the protein in the medium for much longer period (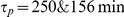). These values of protein half life are significantly higher than what is thought to be the half lives of Bicoid proteins [5]. Further, the source amplitude being so high is inconsistent with the observation that Bicoid protein is often undetectable during the very early stages of development ([5], [19]). Thus, it is reasonable to conclude that the source supply is regulated as suggested by Surdej *et al.*, rather than either kept constant throughout or be subject to natural decay.

**Figure 2:**
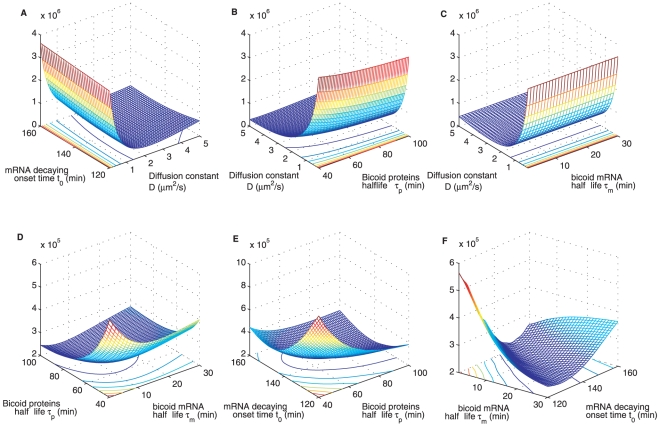
Cross sections through the error function between model output and measurements. Figures show the error function with respect to parameters taken pairwise, with those not shown held constant at their optimum values given in Table 1. Over the parameter ranges considered for the search, the error surface turns out to be unimodal for all three models. Deterministic diffusion model is shown above. Also see Figures S3, S4, S5 for other models considered.

Figure 2 shows cross sections of the error function at the optimum point found by grid search. We have shown this with respect to all parameter combinations, taken pair-wise, setting the parameters not shown to their optimum values. The unimodal form of these error functions, shown here for the deterministic diffusion model, confirms that the optimization strategy we chose was adequate for this purpose. Similar error surface plots for the other two models are given in Figures S3, S4 and S5.

We note that previous authors working on Bicoid profiles have used a range of different values for diffusion and protein half-life parameters. For the diffusion constant, for example, values of, 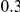 [13], 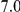 [41]–[43] and 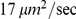 [11], [29] have been used. With our models, we explored the effect of fixing one or more of the parameters at a value used by previous authors and optimizing the remaining parameters. We found the dominant effect is one of the diffusion term compensating for the protein half-life, with the decay onset time and transcript half lives we compute showing far less variation.

**Figure 3:**
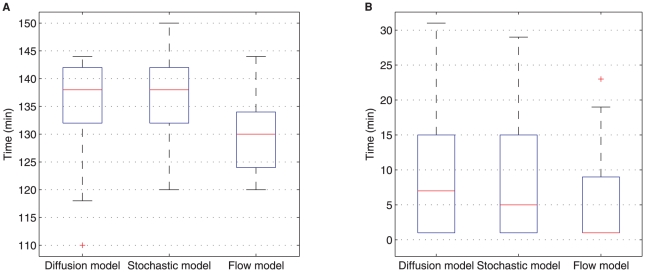
Uncertainty estimation. Uncertainty estimates of mRNA decay onset time 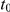 in (A) and degradation time 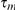 in (B) by fitting the models to 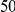 bootstrap samples of individual embryo measurements from FlyEx.

We have further quantified the uncertainties in our estimates of 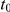 and 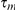 by fitting the models to individual embryo measurements in FlyEx rather than their average profiles. We achieved this by constructing 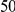 reference datasets by uniformly bootstrapping from each temporal class in FlyEx. Figure 3 shows these uncertainties as box plots and confirms the fact that the estimated onset and decay rates are consistent across all three models.

**Figure 4:**
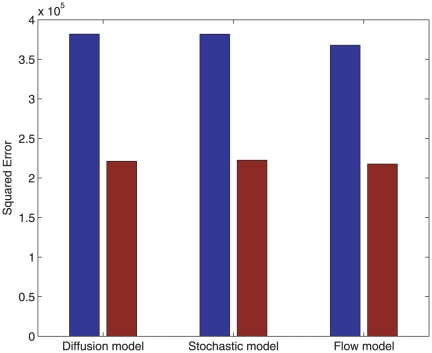
Reduction in squared error between model outputs and FlyEx measurements. In all three models, nearly a factor two reduction is achieved by the improved source whose parameters are optimized. Blue bars represent modelling errors for a constant source model and the red bars correspond to the regulated mRNA model.

Figure 4 shows how the models achieve a reduction of almost a factor two, in the mean squared error between model outputs and FlyEx measurements in the post-peak region of nuclear cleavage cycles 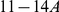. This comparison between modelling errors, with and without our regulated source, confirms the merits of explicitly modelling the destruction of maternally deposited mRNA.

Our models permit the exploration of other published hypotheses about potential mRNA regulation. For example, Salles *et al.* [40], treating the poly(A) tail length of *bicoid* mRNA as proxy for its translational competence, suggest that protein production may be restricted in time, peaking between 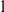 to 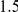 hours in development. We have simulated this by implementing the source as a rectangular function between 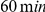 and cycle 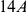, and computing the resulting Bicoid profile (included as Figure S6). We found the corresponding modelling error to be significantly higher, caused mainly by forcing the decay to be instantaneous. While other, similar, explorations are possible with our approach (e.g. polysomal translation and translational bursting [44]), we believe the coarse nature of available data would mean one may have to be cautious about applying models of greater sophistication.

**Figure 5:**
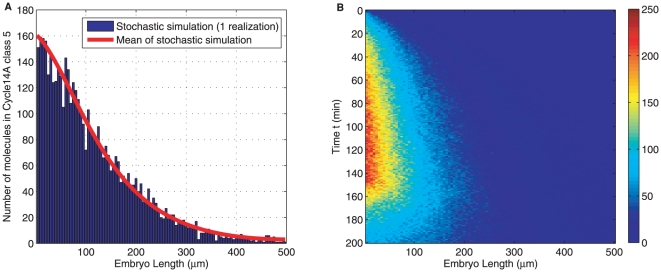
One realization of stochastic simulation by Gillespie algorithm. Blue histogram, (A), shows the numbers of Bicoid molecules along anterior and posterior axis in embryo at a particular time point: cycle 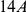 class 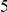. Average of several such simulations is used as model output to match against measurements. (B) shows the realization jointly in space and time.

The results for Bicoid stochastic reaction diffusion in one run of stochastic simulation based on Gillespie algorithm *Direct Method* (Algorithm 1) is shown in Figure 5. This model provides a more detailed understanding of the protein distribution, partitioned in compartments along A-P axis. We note that such a stochastic model characterizes a detailed view arising from molecular level variabilities. Our implementation in deriving the main results for the stochastic model in Table 1, following the technique of Erban *et al.* [45], via simultaneous ordinary differential equations corresponding to discrete bins along the spatial axis (see Materials and Methods), captures average behaviour. Asymptotically (*i.e.* with increasing number of bins), this is the equivalent of averaging a large number of Gillespie simulations, and should also give the same solution as the deterministic differential equation. To estimate the effect of molecular level variation, we matched profiles generated by individual Gillespie simulations to bootstrap samples of Bicoid profiles from FlyEx (the same data used to derive uncertainties in Figure 3). As this process is computationally demanding, we restricted ourselves to estimating the variability in mRNA decay onset time only, with the remaining parameters fixed to their optimal values given in Table 1. Matching such individual simulations to data resulted in an increase in the standard deviation of estimation from 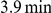 to 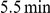. While this increase suggests the variability at the molecular level may be captured by stochastic simulations, as in the study of Wu *et al.*, the resulting estimation uncertainties in both cases are still small for the mRNA decay onset.

### Spatially Distributed *bicoid* mRNA

While nearly all modelling work on Bicoid assume a spatial point source for *bicoid* mRNA, as noted earlier, Spirov *et al.* [27] suggest that the *bicoid* mRNA may have spatial distribution which alone explains the morphogen gradient at the protein level. They argue for an active transport mechanism along a cortical microtubular network. This proposal is questioned by Little *et al.* [41] who show experimental evidence that a distributed spatial gradient of mRNA is not sufficient to achieve the required morphogen profile. Since computational modelling of active transport hypothesized by Spirov *et al.* [27] is outside the scope of this study, we instead follow Dilão *et al.* [46] who have postulated an mRNA diffusion model to achieve an effect similar to that of Spirov *et al.* [27] (see Materials and Methods).

**Figure 6:**
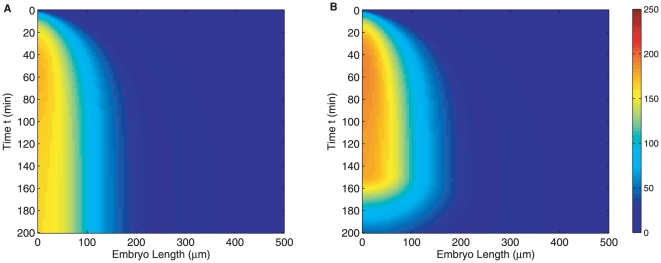
The effect of *bicoid* mRNA spatial gradient. (A) protein intensity without mRNA temporal regulation; (B) Bicoid profile with mRNA temporal regulation.

Figure 6 shows protein intensities with a spatial distribution for *bicoid* mRNA. Figure 6A is profile obtained with only spatially distributed mRNA, while Figure 6B is results obtained with spatial distribution and temporal regulation and the post peak decay is clearly observed. Thus, even with simulated spatial distribution of maternal mRNA, our model finds a set of feasible parameter values that account for observed profiles in FlyEx. The corresponding parameter estimates are shown in Table S2. We find that the differences are in directions we would naturally expect: *i.e.* a spatially distributed maternal mRNA is compensated primarily by faster protein degradation. But it is encouraging to see that the onset of decay (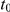) changes only slightly.

## Materials and Methods

### Deterministic Diffusion and Flow Model

The reaction diffusion equation used to model morphogen establishment is given by:
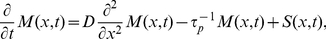
(1)where, 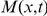 is the morphogen concentration as a spatio-temporal function, 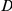, the diffusion constant, 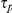, the half-life of the morphogen protein and 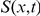, the source at the anterior pole of embryo.

The flow model with one dimension fluid velocity 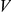 is defined by:
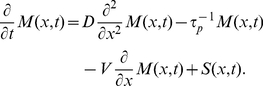
(2)In the original formulation of this model, the flow term was permitted to be active only for a short duration in time: nuclear cleavage cycles 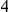 to 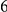 depending on the motion of the nuclei in the viscous cytoplasm. In our implementation, we allowed this term to be present throughout the developmental time period considered, to increase its difference from the standard diffusion model.

The usual assumption in solving these models is that the source is constant: 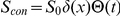, where 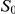 is the production rate, 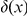 is the Kronecker delta function and 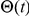 is Heaviside step function. The source model we propose here, incorporating regulated mRNA stability is given by:
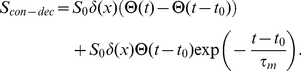
(3)


We use numerical methods available in the MATLAB PDE solver pdepe to solve these reaction diffusion models.

### Stochastic Simulation Model

Closely following Wu *et al.* [21] and Erban *et al.* [45], the stochastic Bicoid protein reaction diffusion system we implemented simulates 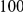 compartments along the A-P axis, each with length 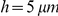, which is approximately the average size of one nucleus.

The three chemical reactions involved in this description are:
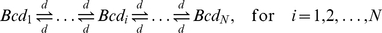
(4)

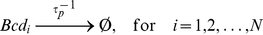
(5)

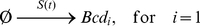
(6)The first of these, Equation (4) describes diffusion between neighbouring sub-volumes, allowed to take place in both directions, at a rate 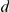, related to the diffusion constant of a deterministic model by 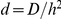. The second, Equation (5), describes protein degradation, and the final, Equation (6), the source. Translation only takes place in the first bin, for 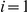.

Our implementation of Gillespie algorithm for stochastic simulation of the master equation closely follows that of Erban *et al.* [45] and is given in pseudo-code format in Algorithm 1. Essentially, this process consists of the generation of two random numbers to select the time at which a reaction occurs, and which one that is. The probability that 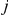-th chemical reaction taking place is given by: 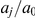, where 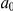 is a total propensity function, computed in step 2 (Algorithm 1).

**Algorithm 1** Bicoid reaction diffusion stochastic simulation.

**Output:** Vector of Bicoid molecular numbers, 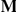


**Initialization:** 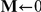, 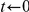


**while** time
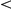
final time **do**

1. Generate two random numbers which are uniformly distributed in 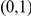: 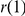 and 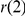.
2. Compute propensity functions for all of the reactions: 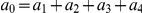.
3. Compute the time when next reaction occurs: 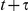, where 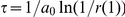.
4. Decide which reaction occurs at 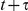: find 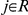 such that: 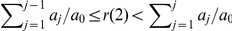.
5. Update numbers of reactants and products in 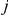-th reaction and set 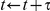.

**end while**

With vector **M**, containing the number of molecules along the 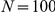 bins, Equations (4)–(6), define a total of 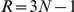 reactions. The propensity functions for the reactions are:
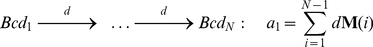
(7)

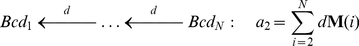
(8)

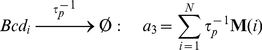
(9)


In order to estimate parameters used in the stochastic model, Gillespie realizations are averaged. Erban *et al.* derive the following system of ODEs for the different compartments to extract the ensemble average directly (see [45] for details):
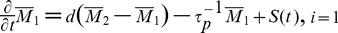
(10)

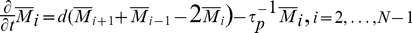
(11)

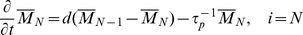
(12)Equations (10)–(12) are solved using MATLAB.

### Matching Models to Data in Joint Space

We use experimental measurements of Bicoid concentrations published in FlyEx database [37], [38] for parameter estimation. FlyEx, providing high resolution quantitative gene expression data by confocal scanning microscopy of fixed embryos, is the best available public domain dataset for this analysis. Measurements published in FlyEx are nuclear concentrations of Bicoid. The models we use, however, correspond to the total Bicoid. We make the assumption that the two concentrations are proportional across the developmental cycles. In recent work, Gregor *et al.* [13] have published some measurements of nuclear and cytoplasmic Bicoid concentrations, showing the dynamical balance between the two during cycles of nuclear division. Their data is suggestive that the use of nuclear concentrations as proxy for total concentrations is reasonable. Once we assume the two are proportional, parameters we infer by matching model outputs and data are unaffected, as any discrepancy will be absorbed by the source amplitude term 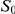, computed by Equation 15.

The spatio-temporal data for Bicoid we use, spans 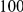 points uniformly spaced along the A-P axis, and covers 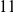 points in time. The temporal range of measurements starts from nuclear cleavage cycle 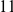 to the end of cycle 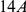. Cycle 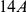 is of specific interest, because it is during this period, cellularization sets in and the established Bicoid profile begins to decay due to the decaying *bicoid* mRNA. While there is some variability in how these developmental stages map onto real time, on average, cycle 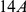 (temporal classes 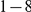) lasts for around 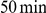 [47]. Cycle 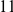 starts around 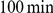 from fertilization, and the three cycles 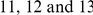 last an average of 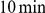 each. In FlyEx, Bicoid data is available for the 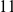 temporal classes from cycle 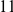 to 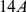.

The squared error between model output and measured intensities is
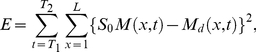
(13)

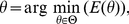
(14)where 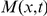 is the model output while 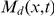 denotes the measured intensities from FlyEx. 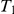 and 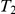 are the boundaries of cleavage cycle 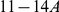. 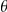 in Equation (14) represents a vector of all unknown model parameters and 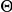 is the space over which we search for optimum values.

Because the model output is linear in the source amplitude 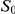 and is independent of the other parameters used in the three models, we calculate it in closed form rather than searching for an optimum in a grid. In order to minimise error 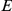 in Equation (13), we differentiate it with respect to 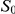 and equate it to zero. Then we have 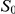 as following:
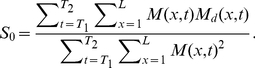
(15)


### *bicoid* mRNA Spatial Distribution

For mRNA spatial distribution, we follow Dilão *et al.*'s work in [46], but do not incorporate a term for natural mRNA decay. Thus instead of a diffusion equation, we restrict ourselves to the heat equation given by:
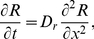
(16)where 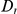 is mRNA diffusion constant. This is justified because our model for the temporal regulation of *bicoid* mRNA is one which holds it stable up to 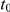 followed by an active degradation.

## Supporting Information

Figure S1 **Spatio-temporal intensity profiles of morphogen concentrations in four different models.** (Ai), solution to deterministic differential equation driven by a constant source (Aii); (Bi), (Ci) and (Di), solutions to deterministic diffusion, stochastic and cytoplasmic flow models, driven by the new source model incorporating regulated mRNA stability, and whose parameters are optimized.
(EPS)

Figure S2
**Spatio-temporal profiles of model outputs and FlyEx data in the regions where model outputs were matched to measured data (nuclear cleavage cycles** 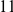 **to** 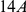 **.** (A), deterministic model driven by a constant source; Models driven by regulated source (C), (D) and (E), are deterministic diffusion, stochastic and cytoplasmic flow respectively. When mRNA regulation is included, all three models faithfully reproduce the temporal decay of the morphogen in the post-peak region.(EPS)

Figure S3 **Modelling error displayed as functions of parameters taken pairwise. Stochastic simulation model.**
(EPS)

Figure S4 **Modelling error displayed as functions of parameters taken pairwise: Cytoplasmic flow model.**
(EPS)

Figure S5 **Modelling error surface for the cytoplasmic flow model as functions of flow velocity parameter and each of the other parameters.**
(EPS)

Figure S6
**Spatio temporal Bicoid profiles with source regulation as a step function, with constant rate of translation between** 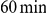 **and onset of cycle** 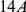 **(implementing** [**40**] **).**
(EPS)

Table S1 **Parameter optimization on a regular grid.** Table S1 shows the search spaces used in optimising the parameters of the three models considered. We used a coarse grid in the first round to get a rough estimate of the sensible range of parameters and followed it with a second round of search with a higher resolution and a reduced search range. Such a strategy is feasible, given we have only five parameters to estimate. Further, given the noisy nature of available data, searching over a finer grid to optimize parameters to a higher level of numerical precision does not make sense. If data of higher quality becomes available in the future, a scheme based on simulated annealing or population based optimisation needs to be considered. With the grid sizes we chose, shown in Table S1, it was possible to do least squares fitting of all three models on a desktop PC, with at most three days of wall clock time.
(PDF)

Table S2 **Parameter estimation for stochastic model with** ***bicoid*** **mRNA regulation and spatial distribution.**
(PDF)
