# Supplementary material for: The Role of Regulated mRNA Stability in Establishing Bicoid Morphogen Gradient in Drosophila Embryonic Development
Source: PLoS One. 2011 Sep 16;6(9):e24896. doi: 10.1371/journal.pone.0024896 (PMC3174985; doi:10.1371/journal.pone.0024896)
Supplement: Table S1 — Parameter optimization on a regular grid. Table S1 shows the search spaces used in optimising the parameters of the three models considered. We used a coarse grid in the first round to get a rough estimate of the sensible range of parameters and followed it with a second round of search with a higher resolution and a reduced search range. Such a strategy is feasible, given we have only five parameters to estimate. Further, given the noisy nature of available data, searching over a finer grid to optimize parameters to a higher level of numerical precision does not make sense. If data of higher quality becomes available in the future, a scheme based on simulated annealing or population based optimisation needs to be considered. With the grid sizes we chose, shown in Table S1, it was possible to do least squares fitting of all three models on a desktop PC, with at most three days of wall clock time. (PDF) [file pone.0024896.s007.pdf]

Table S1. Parameter optimization on a regular grid.

Space and resolution for the first round of search.			
Parameters	Diffusion model	Stochastic model	Flow model
$D(\mu m^2/s)$	0.1:0.2:5	0.1:0.2:5	0.1:0.3:5
$t_0(min)$	120:2:160	120:2:160	120:3:160
$\tau_p(min)$	40:2:100	40:2:100	40:3:100
$\tau_m(min)$	1:2:30	1:2:30	1:3:30
$V(\mu m/s)$	Null	Null	0.01:0.02:0.1
Space and resolution for the second round of search.			
Parameters	Diffusion model	Stochastic model	Flow model
$D(\mu m^2/s)$	2:0.1:4	2:0.1:4	0.1:0.1:1.5
$t_0(min)$	135:1:150	135:1:150	135:1:150
$\tau_p(min)$	70:1:95	70:1:95	35:1:50
$\tau_m(min)$	1:1:15	1:1:15	1:1:12
$V(\mu m/s)$	Null	Null	0.02:0.01:0.06
