# Supplementary material for: The Role of Regulated mRNA Stability in Establishing Bicoid Morphogen Gradient in Drosophila Embryonic Development
Source: PLoS One. 2011 Sep 16;6(9):e24896. doi: 10.1371/journal.pone.0024896 (PMC3174985; doi:10.1371/journal.pone.0024896)
Supplement: Table S2 — Parameter estimation for stochastic model with bicoid mRNA regulation and spatial distribution. (PDF) [file pone.0024896.s008.pdf]

Table S2. Parameter optimization for stochastic simulation model with mRNA regulation and spatial distribution.

Parameter	$D(\mu m^2/s)$	$t_0(min)$	$\tau_p(min)$	$\tau_m(min)$	$D_r(\mu m^2/s)$	S_0
	3.7	148	30	19	0.4	170
